# Myxoid dermatofibrosarcoma protuberans of the foot: Clinicopathologic and dermatoscopic features

**DOI:** 10.1016/j.jdcr.2026.07.063

**Published:** 2026-08-07

**Authors:** Mariana Guadalupe Ungson García, Ángel Iván De Dios Aceves, Mariana Ruiz León, Nidia Guadalupe Aguilar Figueroa, Martha Alicia Aceves Villalvazo

**Affiliations:** a Private practice, Hermosillo, Sonora, Mexico; b Centro Universitario de Ciencias de la Salud, Universidad de Guadalajara, Guadalajara, Jalisco, Mexico; c Hospital Regional "Dr Valentín Gómez Farías", Zapopan, Jalisco, México; d Hospital General Regional No. 180, Instituto Mexicano del Seguro Social (IMSS), Tlajomulco de Zúñiga, Jalisco, Mexico

**Keywords:** CD34, dermatofibrosarcoma protuberans, fibroblastic, histopathology, myxoid

## Introduction

Dermatofibrosarcoma protuberans (DFSP) is a rare, locally aggressive, fibroblastic cutaneous tumor with high local recurrence rate.^1^, ^2^, ^3^ It represents 1% to 2% of all soft tissue sarcomas. The annual reported incidence in the United States ranges from 4.2 to 5.0 cases per million person-years, usually in patients aged between 30 and 40 years.^4^ DFSP typically involves the trunk (42%-52.7%), proximal upper (12.3%-35.4%) and lower extremities (6.8%-12.7%), and head and neck (10.6%-17.5%), whereas involvement of distal extremities is uncommon. DFSP is usually asymptomatic and slow growing. It presents as an indurated, erythematous, or yellowish-brown mass with irregular borders, ranging from 2 to 5 cm.^5^^,^^6^ Histopathologic examination is definitive for establishing the diagnosis. Surgery is the treatment of choice, and metastasis risk is low (2%-5%).^4^^,^^5^ This case emphasizes the importance of dermatoscopic findings, histologic diagnosis, and complete tumor excision.

## Case report

A 66-year-old patient with gout managed with allopurinol presented to the dermatology service for evaluation of a 10-year-old right foot nodule. After trauma, the lesion became painful, bled, and grew rapidly. Physical examination revealed an exophytic well-circumscribed mobile nodule measuring 8 × 6 × 7 cm without fixation to deeper structures. Prominent blood vessels were noted at the base and along the surface, which was partially covered by a serosanguineous crust (Fig 1). Dermatoscopy revealed linear and serpentine vessels over a heterogeneous reddish background, white and yellowish structureless areas (Fig 2). Lower extremity and chest contrast computed tomography scan showed no evidence of metastasis or deep infiltration. A deep incisional biopsy ruled out DFSP, melanoma, or leiomyosarcoma. Histopathologic and immunohistochemical examination (Fig 3) showed a spindle cell mesenchymal tumor with a prominent myxoid component involving >50% of the lesion, branching small thin-walled vessels, and a preserved storiform architecture. Notably, honeycomb infiltration of the subcutis was not prominent. Cells exhibited mild atypia and pleomorphism, with a low mitotic rate. No evidence of lymphovascular invasion was identified. Diffuse strong positivity for CD34 and negativity for S100 was demonstrated. A diagnosis of myxoid DFSP was made (M-DFSP). Mohs micrographic surgery was unavailable; therefore, a wide local excision (WLE) was performed with 2.5-cm lateral and 1-cm deep margins, including the fascia (Fig 4). Histopathologic analysis confirmed negative margins. No recurrence was observed during 5 years of follow-up.

**Fig 1:**
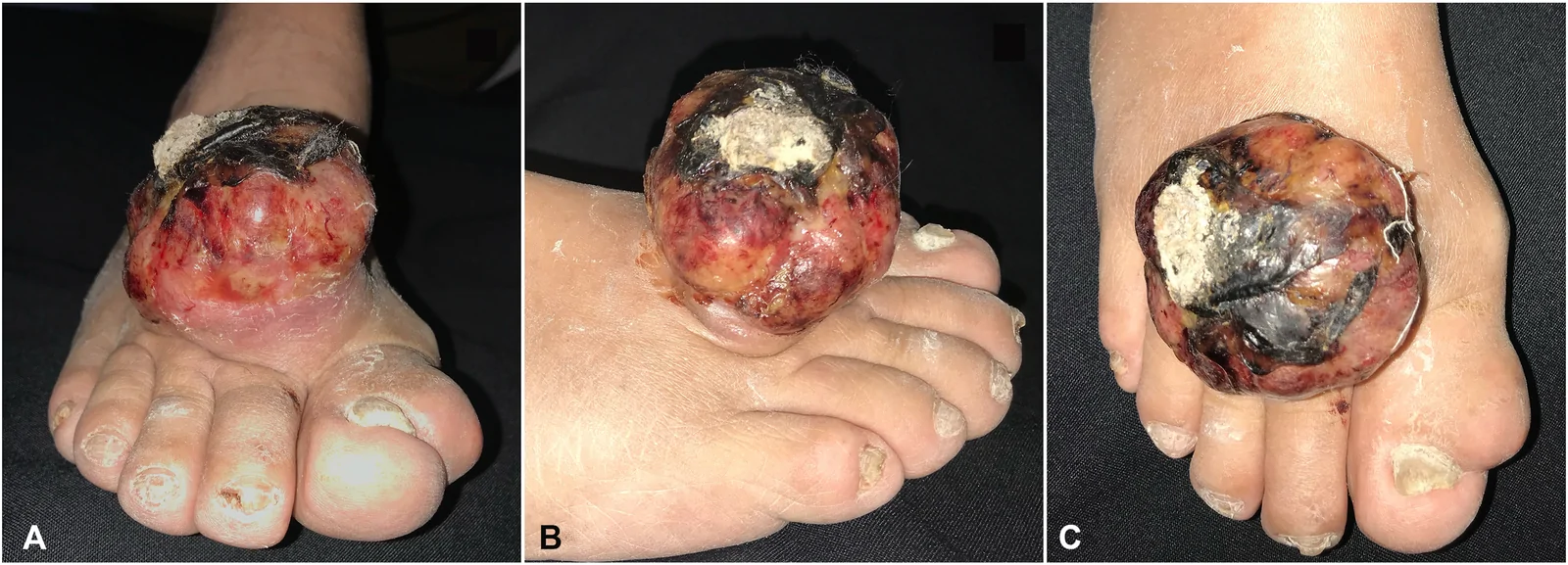
Dorsum aspect of the foot with an exophytic nodular lesion reddish-violet with multiple blood vessels at the base and along the nodule, covered by a crust. **A,** Frontal view. **B,** Sagittal view. **C,** Axial view, tophus is observed on the medial surface of the first finger.

**Fig 2:**
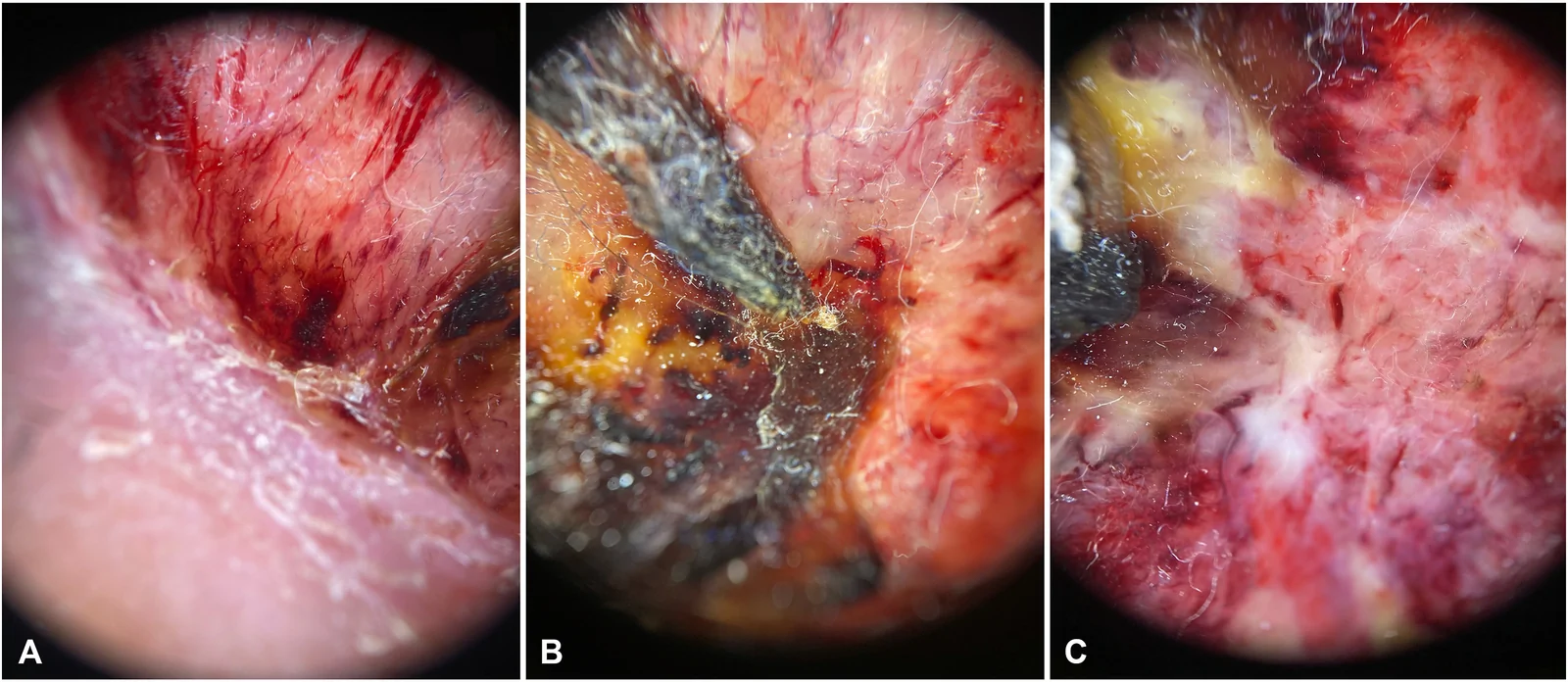
Dermatoscopy images show a nonmelanotic lesion where the following are observed: **A,** Arborizing and linear thin and thick irregular vessels on the base of the nodule. **B,** Red-yellowish crust, curved and linear vessels. **C,** Red, yellow, and white structureless areas, irregular linear and curved vessels.

**Fig 3:**
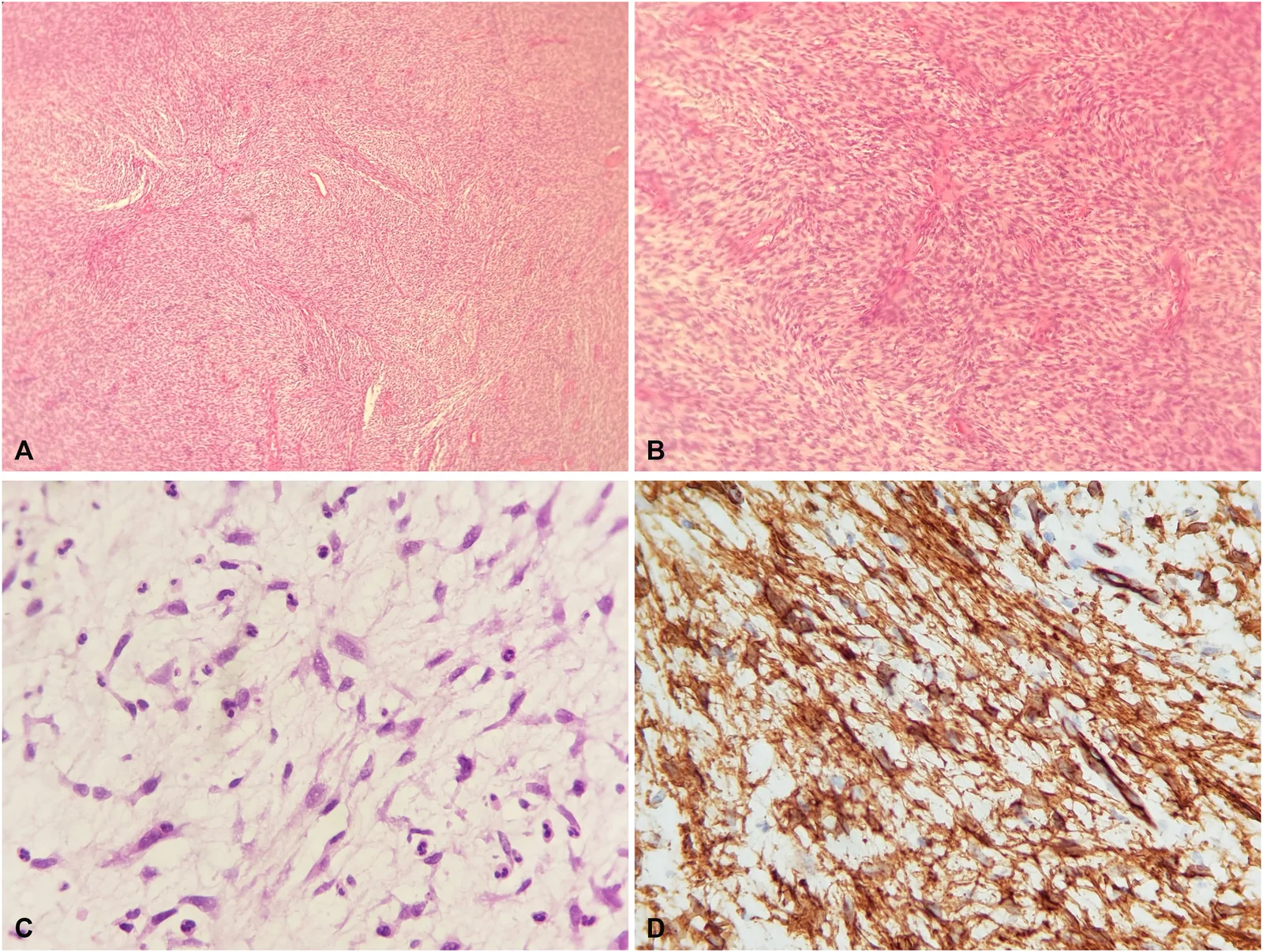
Histopathology of myxoid dermatofibrosarcoma protuberans. **A,** The hematoxylin-eosin–stained section at low power illustrates a dermal tumor composed of spindle-shaped cells. **B,** A myxoid background is identified. **C,** At high power, cells show mild atypia. **D,** CD34 immunostaining reveals diffuse positivity.

**Fig 4:**
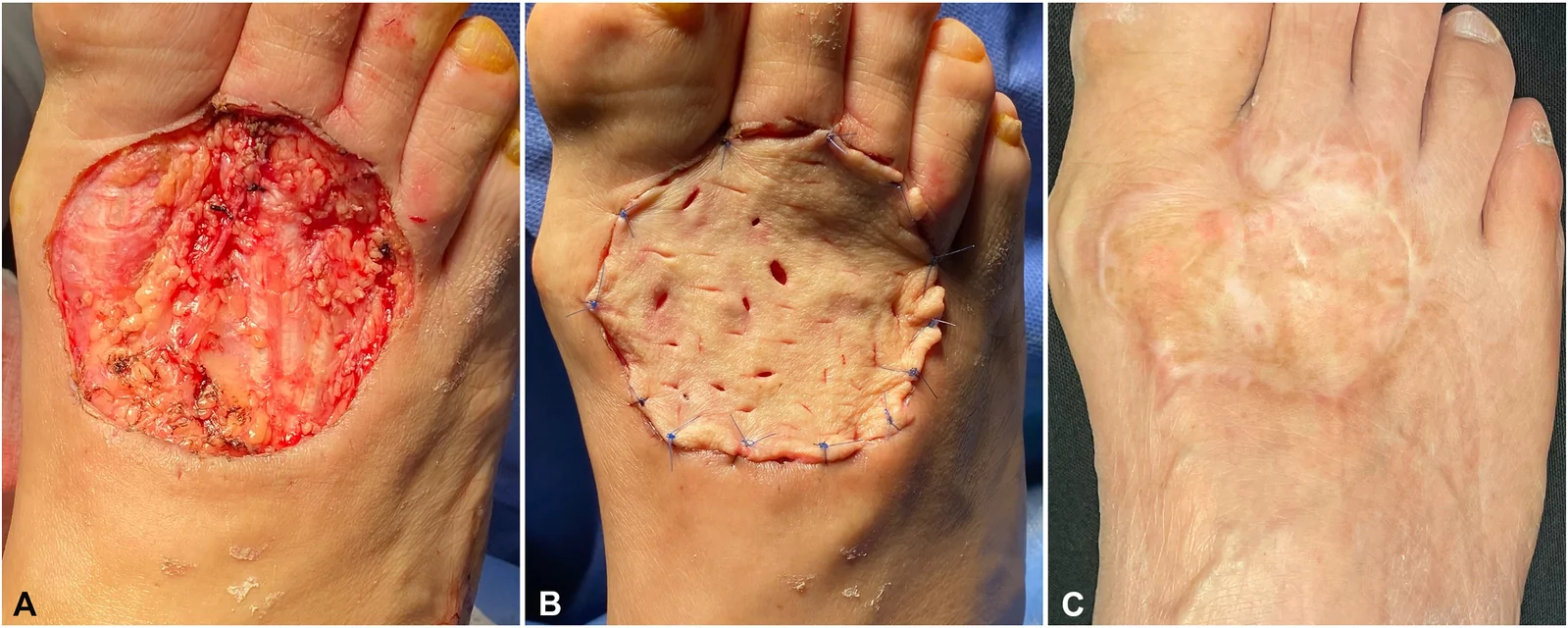
A, Nodule removed by wide local excision. **B**, Placement of autograft skin in the affected area. **C**, 5-year follow-up, showing a depigmented asymptomatic scar with no evidence of recurrence.

## Discussion

DFSP is often misdiagnosed or diagnosis is delayed due to its low incidence, slow progression, and nonspecific early clinical features. An adequate tissue sample obtained from punch or deep incisional biopsy reaching the subdermis is required.^1^ DFSP is a dermal neoplasm composed of spindle cells arranged in a storiform pattern with minimal cytologic atypia and low or absent mitotic activity.^3^ Subdermal infiltration is diffuse, creating a honeycomb pattern usually with tentacle-like extensions.^5^ Other histologic variants are M-DFSP, pigmented, myoid, granular cell, sclerosing, atrophic, giant cell fibroblastoma, and DFSP with fibrosarcomatous transformation.^4^^,^^7^ The myxoid variant is uncommon. It behaves similarly to classic DFSP but shows a distinct predilection for the extremities.^8^ It is composed of spindle cells with pale cytoplasm arranged in lobules. A myxoid stroma, which must be present in >50% of the tumor, may obscure the typical storiform pattern^2^^,^^7^ showcasing prominent branching vessels. Classic honeycomb infiltration and diffuse and strong CD34 positivity remain preserved. Mitotic figures are scarce and lack cytologic atypia.^5^^,^^7^ M-DFSP must be distinguished from other myxoid neoplasms. Superficial angiomyxoma features prominent neutrophils and CD34 heterogeneity rather than diffuse strong positivity.^2^^,^^5^^,^^7^ Myxofibrosarcoma exhibits nuclear pleomorphism, atypical mitoses, and curvilinear elongated vessels, typically lacking diffuse CD34 expression.^3^^,^^8^ Myxoid neurofibroma demonstrates S100/SOX10 positivity but CD34 negativity.^7^ Spindle cell melanoma was ruled out by the absence of S100 expression, atypical junctional component, atypical mitoses or necrosis.^3^^,^^7^ Solitary fibrous tumor can share CD34 positivity but it typically presents a patternless architecture with prominent hemangiopericytoma-like staghorn vessels.^3^ Lastly, DFSP with fibrosarcomatous has a larger tumor size, rapid growth, and pain.^7^ Usually, it shows a herringbone pattern, increased cellularity, cytologic atypia, elevated mitotic activity, and usually loss of CD34 immunoreactivity.^5^^,^^7^ Although molecular or cytogenetic analysis was not performed because of limited resources, the clinical, histopathologic, and immunohistochemical features strongly confirm the diagnosis of M-DFSP.^3^^,^^8^

Only a limited number of cases have described the dermatoscopic features of DFSP. Common findings include a delicate pigment network, peripheral mesh-like vessels, shiny white streaks, and structureless white areas correlating with hyperpigmented basal layer, dilated vessels in the papillary dermis, collagen deposits or fibrosis of interlacing bands of tumor cells arranged in a storiform pattern, and dermal atrophy. Additionally, yellowish structureless areas may reflect infiltrative extension into the subcutaneous tissue correlating with the honeycomb pattern.^9^ Overall, the lesion exhibits a multicomponent dermatoscopic pattern, which is highly indicative of malignancy.^5^^,^^9^ Despite the limited dermatoscopic data on the myxoid variant, the features observed in this case were aligned with those reported in other variants.^9^

According to American and European guidelines, the treatment of choice is excision with Mohs micrographic surgery or peripheral and deep en face margin assessment. If unavailable, the main alternative is WLE.^1^^,^^5^ An inadequate initial excision frequently leads to large, deeply recurrent lesions.^9^ European guidelines recommend lateral margins of ≥2 to 3cm along with resection of the underlying fascia.^5^ Recurrence risk ranges from 0% to 6.6% with Mohs micrographic surgery and 1.7% to 30.8% with WLE. However, WLE combined with complete margin evaluation achieves a 1% recurrence rate, emphasizing meticulous margin assessment as the most important prognostic factor.^1^^,^^5^ Follow-up is recommended every 6 months during the first 3 years and annually for 2 additional years. Shorter evaluation or longer intervals are advised for patients with high-risk features.^5^

DFSP requires a high index of suspicion and a multidisciplinary approach for optimal outcomes. We highlight this case because of the rarity of the myxoid variant and its clinical and dermatoscopic features, which required exclusion of myxoid neoplasms in a patient whose prior long-standing asymptomatic and slow-growing lesion developed pain, rapid growth, and bleeding after trauma.

### Declaration of Generative AI and AI-Assisted Technologies in the Writing Process

None.

## Conflicts of interest

None disclosed.
